# Supplementary material for: Cell adhesion molecule KIRREL1 is a feedback regulator of Hippo signaling recruiting SAV1 to cell-cell contact sites
Source: Nat Commun. 2022 Feb 17;13:930. doi: 10.1038/s41467-022-28567-3 (PMC8854406; doi:10.1038/s41467-022-28567-3)
Supplement: Supplementary file 1 — Supplementary Information [file 41467_2022_28567_MOESM1_ESM.pdf]

Cell adhesion molecule KIRREL1 is a feedback regulator of Hippo signaling recruiting SAV1 to cell-cell contact sites

Atanu Paul[1], Stefano Annunziato[2], Bo Lu[1], Tianliang Sun[2], Olivera Evrova[2], Lara Planas-Paz[2], Vanessa Orsini[2], Luigi M. Terracciano[3,4], Olga Charlat[1], Zinger Yang Loureiro[1], Lei Ji[1], Raffaella Zamponi[1], Frederic Sigoillot[1], Hong Lei[1], Alicia Lindeman[1], Carsten Russ[1], John S. Reece-Hoyes[1], Thomas B. Nicholson[1], Jan S. Tchorz[2], Feng Cong[1*]

List of Supplementary Items:

Supplementary Figures with Legends 1-9

Supplementary Tables 1-3

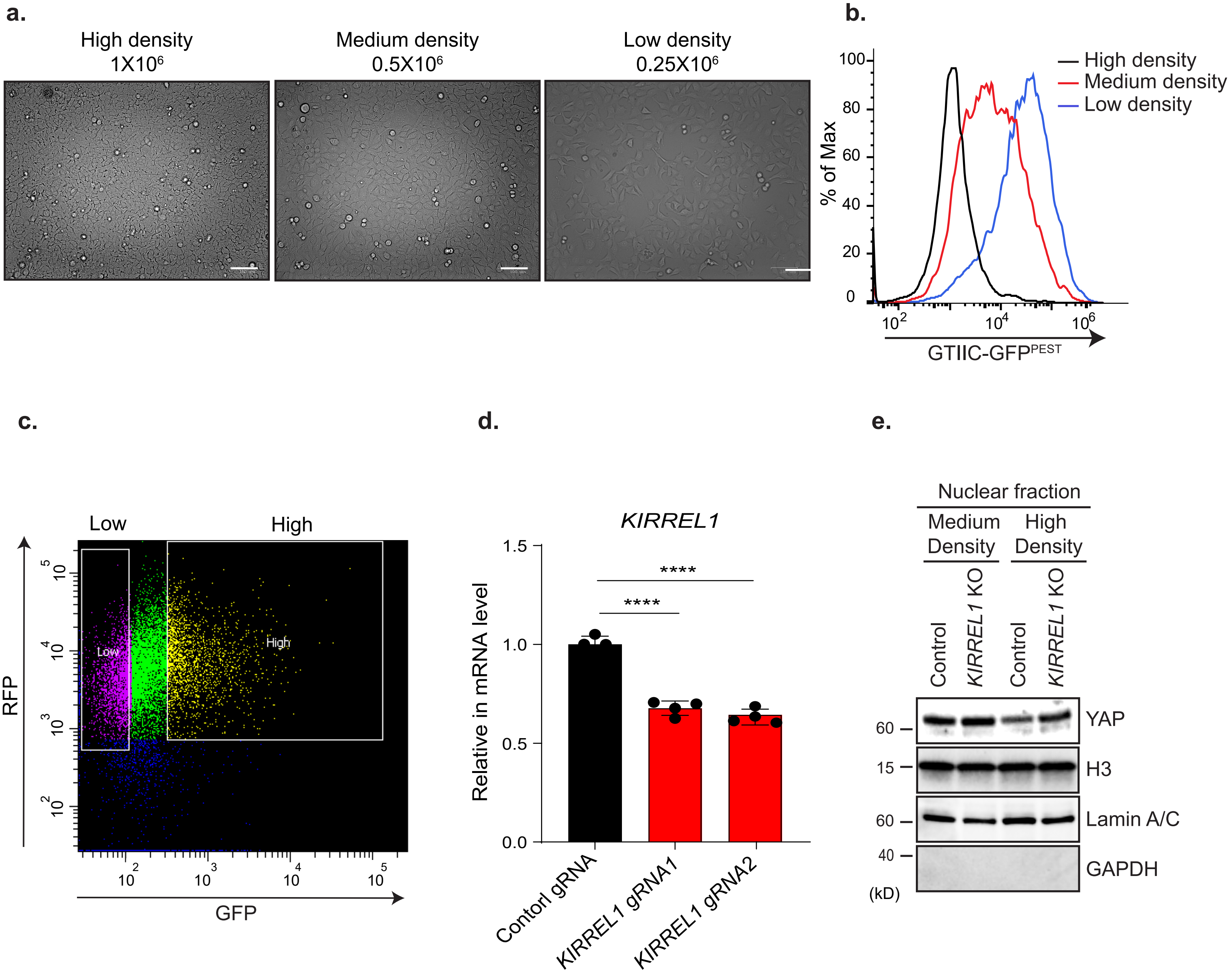

Figure S1. Identification of KIRREL1 as a positive regulator of Hippo signaling from CRISPR screen.
a. Representative bright-field images showing cell density of HEK293A GTIIC-GFP^{PEST} Cas9 cells plated at high, medium, or low density 24 hrs post cell plating. Scale bar, 100 μm. **b.** Density-dependent regulation of GTIIC-GFP^{PEST} in HEK293A GTIIC-GFP^{PEST} Cas9 cells. Cells were plated at high, medium, or low density and GTIIC-GFP^{PEST} reporter activity was assessed by flow cytometry. **c.** Raw FACS data showing lentiviral library transduced RFP-positive HEK293A GTIIC-GFP^{PEST} Cas9 cells sorting into GFP-high and GFP-low populations. Since gRNA expressing lentivirus also expresses gRNA and RFP-T2A-Puro, the majority of cells are RFP positive. **d.** Knockout of *KIRREL1* by independent gRNAs decreases the expression of *KIRREL1* mRNA in HEK293A cells. Frameshift mutation induced by *KIRREL1* gRNA reduces the expression of *KIRREL1* mRNA through nonsense-mediated decay. Data represent mean ± SD, n=4, error bars denote the SD between four biological replicates; Unpaired two-tailed *t*-test was used to determine the statistical significance, ****p value < 0.0001. **e.** Knockout of *KIRREL1* increases the expression of YAP protein in the nuclear fraction. Control or *KIRREL1* KO HEK293A cells were plated at indicated densities in12-well plate and nuclear extractions were prepared 24 hrs post cell plating and subjected to Western blot analysis with indicated antibodies. Histone H3 and Lamin A/C were used as loading control of nuclear proteins. Cytoplasmic protein GAPDH was used as control for nuclear fractionation. Source data for Fig. S1d and e are provided as Source Data file.

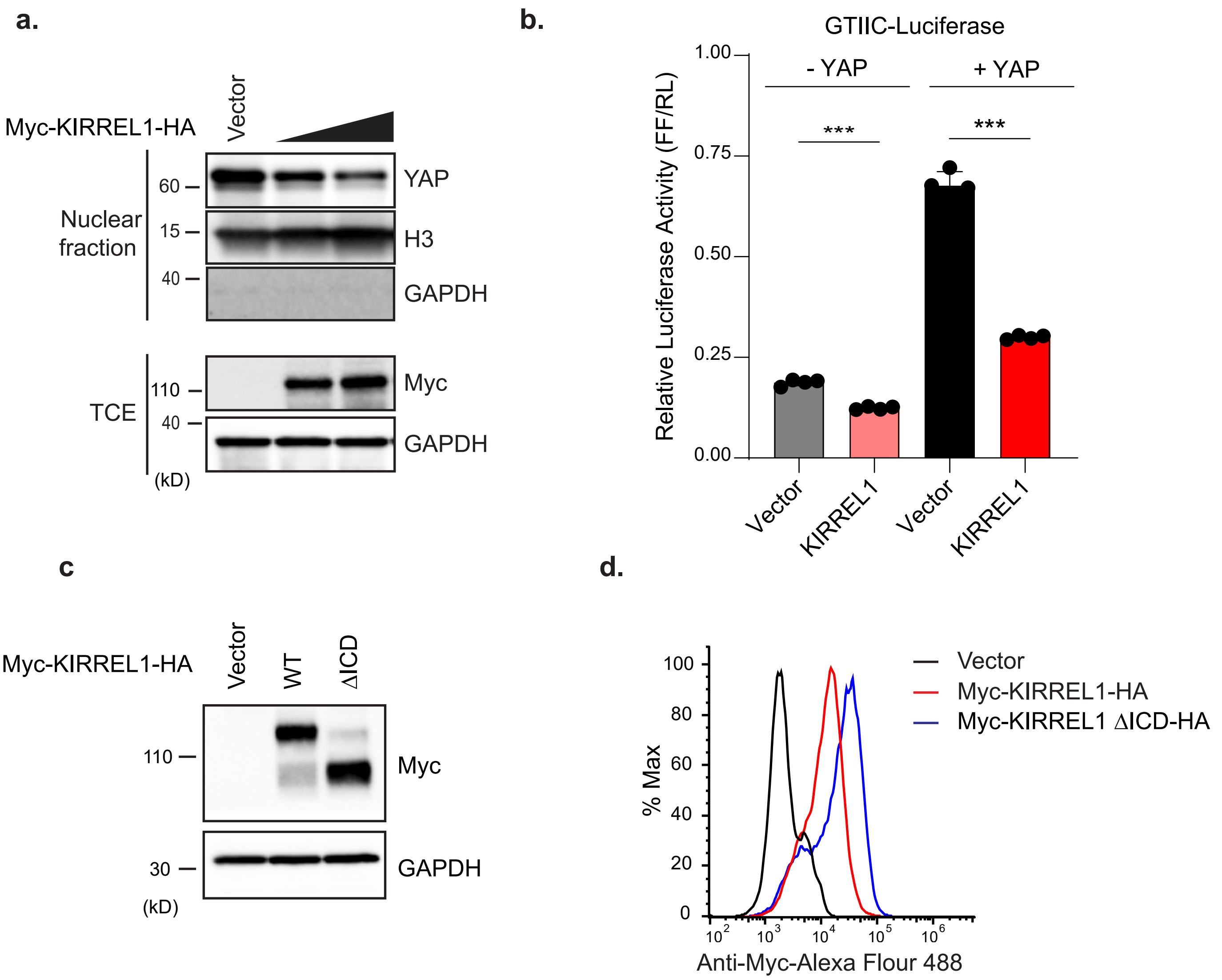

Figure S2. KIRREL1 ICD is required for KIRREL1-mediated inhibition of YAP activity. a. Overexpression of KIRREL1 decreases nuclear accumulation of YAP. HEK293A cells transiently transfected with increasing amounts of Myc-KIRREL1-HA and nuclear fractions were isolated 24 hours-post transfection and subjected to Western blot a nalysis with indicated antibodies. **b.** Overexpression of wild-type KIRREL1 suppresses GTIIC luciferase reporter activity. HEK293A cells were co-transfected with YAP and indicated constructs along with GTIIC-luciferase and TK-Renilla luciferase and subjected to dual luciferase assay. Data represent mean ± SD, n=4, error bars denote the SD between four biological replicates; Unpaired two-tailed *t*-test was used to determine the statistical significance, ***p value < 0.001, ns – 0.4826. **c.** Western blot analysis of WT and ΔICD mutant of KIRREL1 in HEK293A cells. HEK293A were transiently transfected with plasmid encoding Myc-tagged wild-type (WT) KIRREL1 or ΔICD mutant and cell lysates were analyzed by Western blot. **d.** Flow cytometry analysis of wild-type KIRREL1 and ΔICD mutant on the cell surface. HEK293A overexpressing Myc-tagged wild-type (WT) KIRREL1 or ΔICD mutant were stained with anti-Myc-Alexa Flour 488 antibody and analyzed by flow cytometry. Source data for Fig. S2a, b and c are provided as Source Data file.

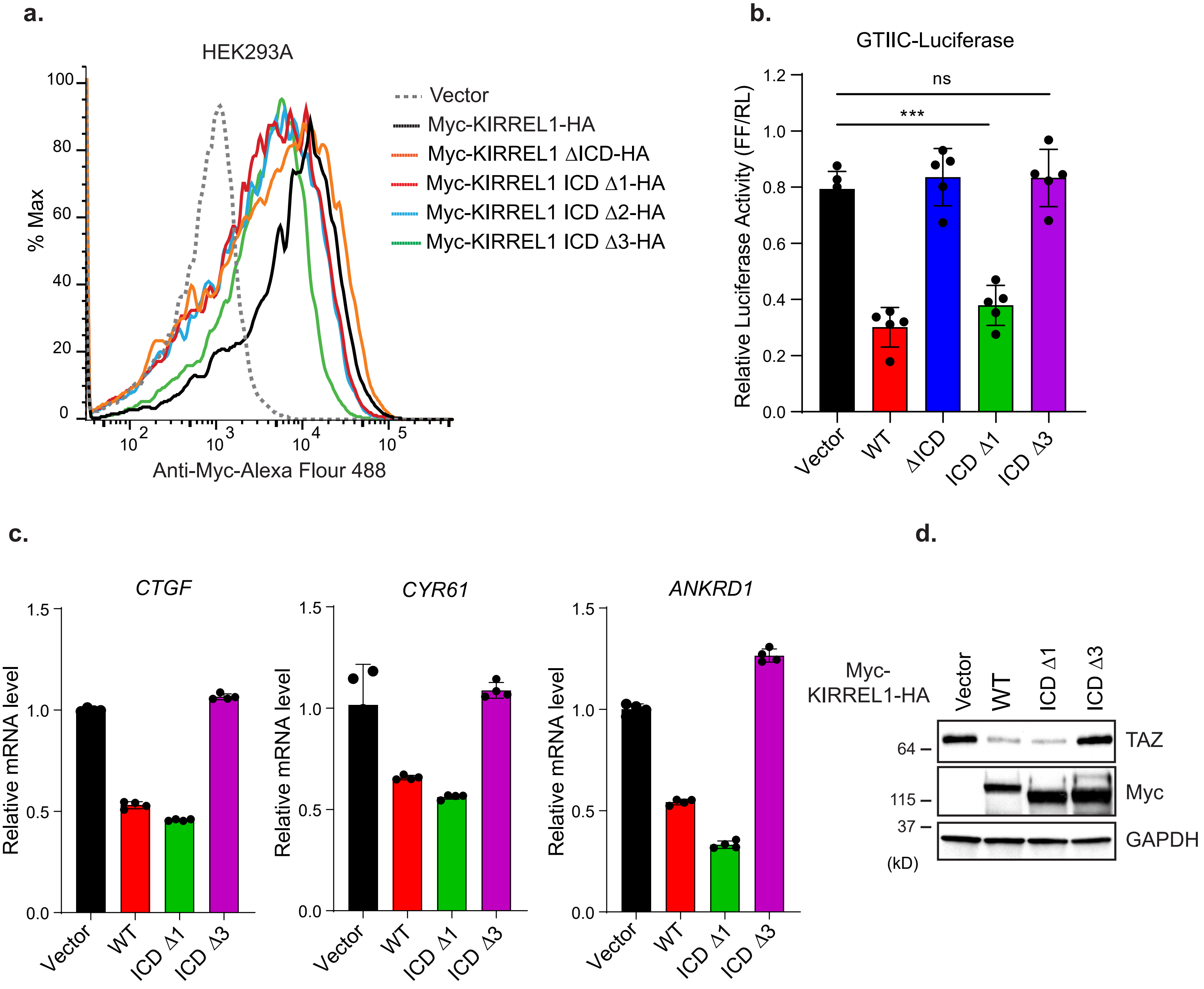

Figure S3. The C-terminus of KIRREL1 is required for KIRREL1-mediated YAP inhibition.
a. Flow cytometry analysis of expression of wild-type and deletion mutants of KIRREL1 on the cell surface. HEK293A cells expressing Myc-tagged WT or deletion mutants of KIRREL1 were stained with anti-Myc-Alexa Flour 488 antibody and analyzed by flow cytometry. **b.** Overexpression of WT KIRREL1, but not ICD Δ3 mutant, decreased expression of GTIIC-Luciferase reporter. Experiment was performed as in Fig. 3g. Data represent mean ± SD, n=4, error bars denote the SD between four biological replicates; Unpaired two-tailed *t*-test was used to determine the statistical significance ***p value < 0.001. **c.** Overexpression of WT, ICD Δ1, but not ICD Δ3, decreased expression of YAP target genes. HEK293A cells overexpressing WT or mutants of KIRREL1 were plated at medium density (0.5X10^6 cells/well) in 12-well plate and analyzed by qRT-PCR 48 hrs post-cell plating. Data represent mean ± SD, n=4. **d.** Overexpression of WT, ICD Δ1, but not ICD Δ3, decreased expression of endogenous TAZ proteins. HEK293A were transiently transfected with indicated plasmids and cell lysates were analyzed by Western blot. Source data for Fig. S3b, c and d are provided as Source Data file.

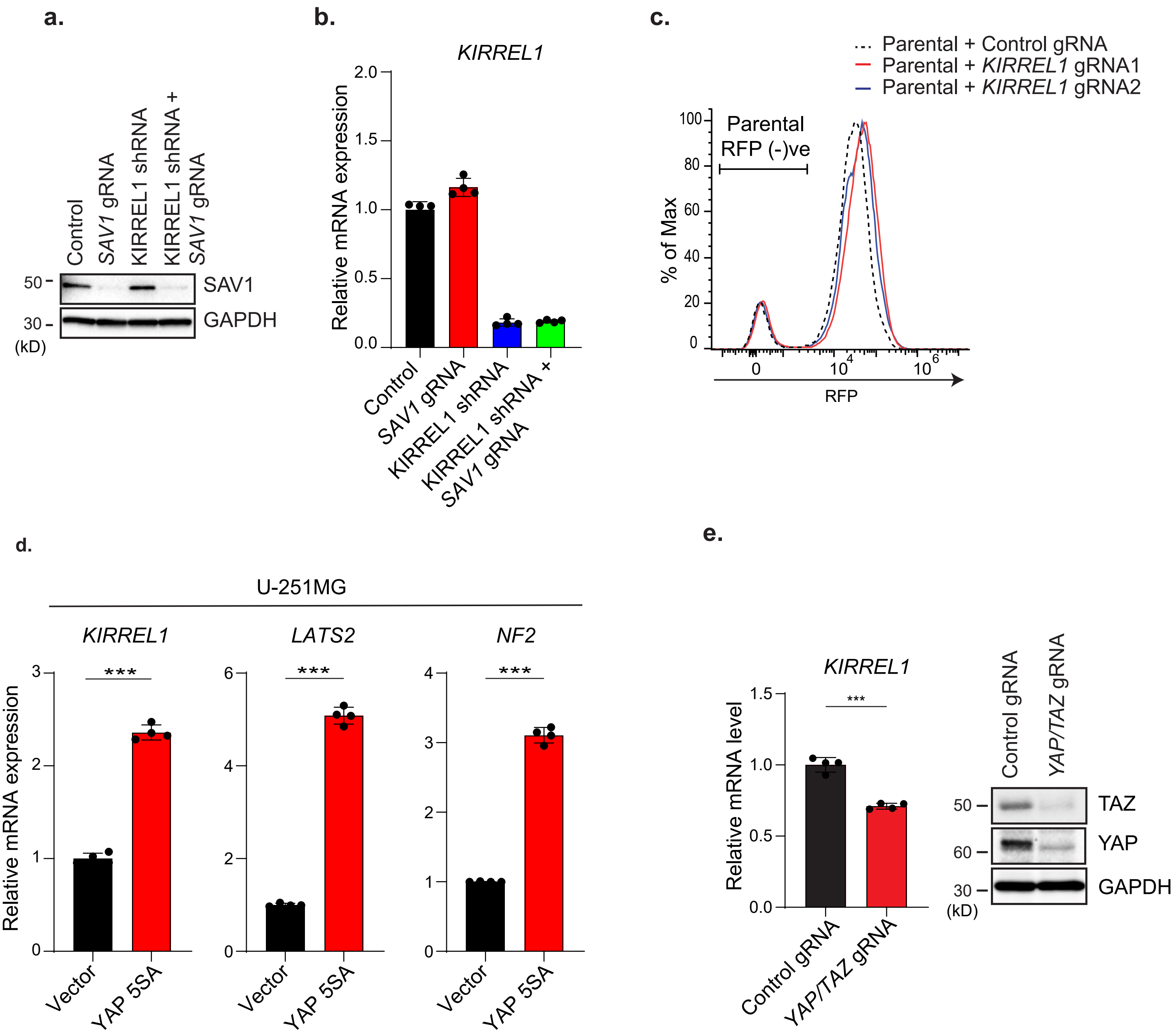

Figure S4. Expression of KIRREL1 is induced by YAP/TAZ. a. Western blot analysis of SAV1 expression in *SAV1* knockout cells. **b.** Quantitative RT-PCR analysis of *KIRREL1* knockdown efficiency. The data represents mean ± SD, n=4 biological replicates. **c.** KIRREL1 suppresses YAP activity in neighboring cells in co-culture assay. HEK293A GTIIC-GFP^{PEST} Cas9 cells (parental) and HEK293A GTIIC-GFP^{PEST} cells transduced with lentivirus expressing RFP and control or *KIRREL1* gRNAs were mixed at 1:10 ratio, co-cultured for 2 days, and analyzed by FACS assay. Plot of RFP signal of co-cultured cells is shown. Parental cells are RFP negative. **d.** Overexpression of constitutively active YAP increases expression of *KIRREL1* in U251-MG glioblastoma cell line. U251-MG cells stably overexpressing YAP 5SA were subjected to qRT-PCR analysis. *LATS2* and *NF2* were used as positive control. Data represents mean ± SD, n=4, error bars denote the SD between four biological replicates; Unpaired two-tailed *t*-test was used to determine the statistical significance ***p value < 0.001. **e.** Double knockout of *YAP/TAZ* decreases expression of *KIRREL1*. HEK293A Cas9 cells transduced with lentivirus expressing control gRNA or *YAP/TAZ* gRNA were subjected to qRT-PCR analysis (left panel). Data represent mean ± SD, n=4, error bars denote the SD between four biological replicates; Unpaired two-tailed *t*-test was used to determine the statistical significance, ***p value < 0.001. Knockout of *YAP* and *TAZ* was confirmed by Western blot analysis (right panel). Source data for Fig. S4a, b, d and e are provided as Source Data file.

a.

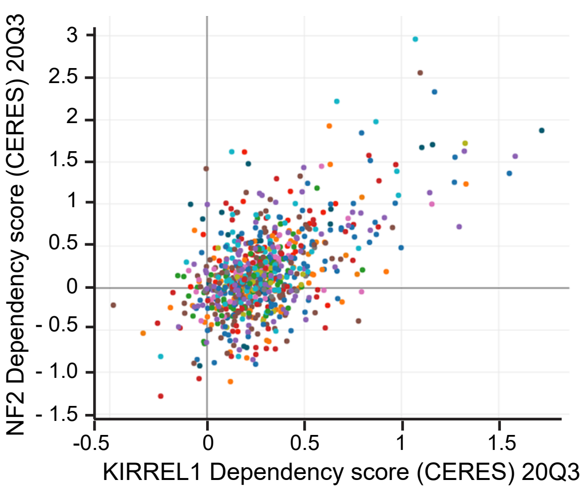

b.

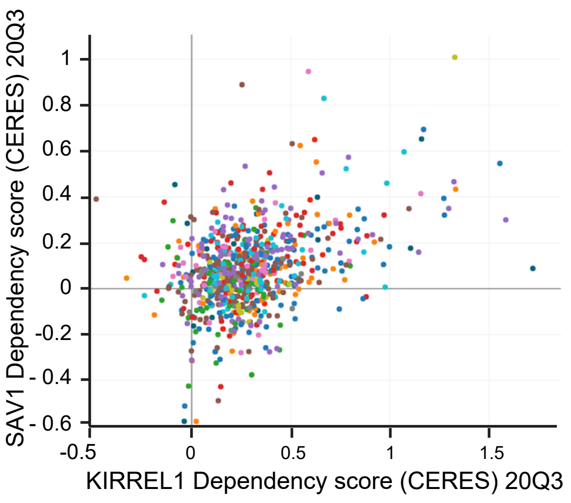

c.

***KIRREL2* Top Co-dependencies**

CRISPR (Avana) 20Q3

Gene	Pearson correlation
FAM187B	0.27
ZNF599	0.27
MAG	0.26
FAM98C	0.25
ZNF576	0.25
ACP7	0.25
C19orf12	0.24
HCST	0.23
RYR1	0.23
IRF2BP1	0.23
COX7A1	0.23

d.

***KIRREL3* Top Co-dependencies**

CRISPR (Avana) 20Q3

Gene	Pearson correlation
AATK	0.18
S100PBP	0.17
GPR108	0.15
PGAP2	0.15
CDK3	0.15
CEP83	0.15
ANTKMT	0.15
SCX	0.15
ZNF695	0.15
TBCEL	0.15
SMIM1	0.15

e.

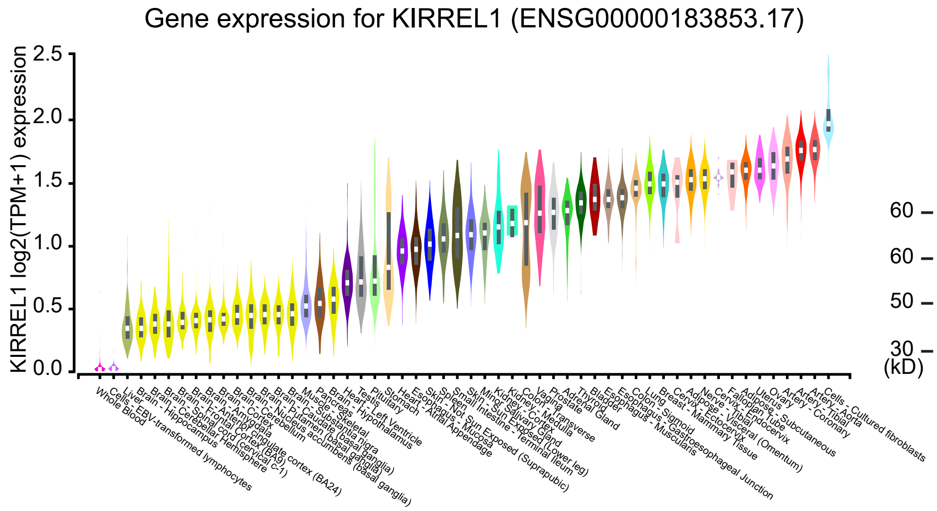

f.

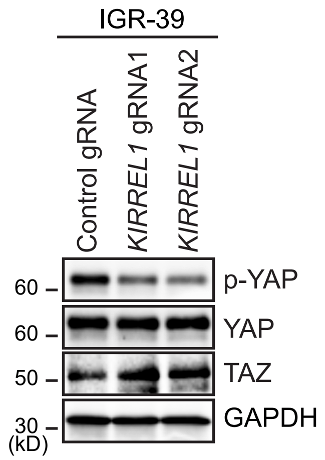

Figure S5. DepMap analysis of gene co-dependency in CRISPR proliferation screens a. Correlation between *NF2* and *KIRREL1* in Project Achilles. CERES scores of *NF2* and *KIRREL1* in CRISPR proliferation screens are plotted. **b.** Correlation between *SAV1* and *KIRREL1* in Project Achilles. CERES scores of *SAV1* and *KIRREL1* in CRISPR proliferation screens are plotted. **c.** Top *KIRREL2* correlated genes in Project Achilles. Pearson correlation score on the right indicates the strength of co-dependency. **d.** Top *KIRREL3* correlated genes in Project Achilles. Pearson correlation on the right indicates the strength of co-dependency. **e.** Tissue-specific gene expression of human *KIRREL1* from GTEx database (https://gtexportal.org/home/gene/KIRREL). mRNA expression values are shown in log TPM (transcripts per million). Box plots are shown as median and 25th and 75th percentiles. Values above or below 1.5 times the interquartile range were considered as outliers and were not shown. **f.** Immunoblot analysis of phospho-YAP, total YAP, and TAZ proteins in IGR-39 cells expressing control or *KIRREL1* gRNAs. GAPDH was used as loading control. Source data for Fig. S5f are provided as Source Data file.

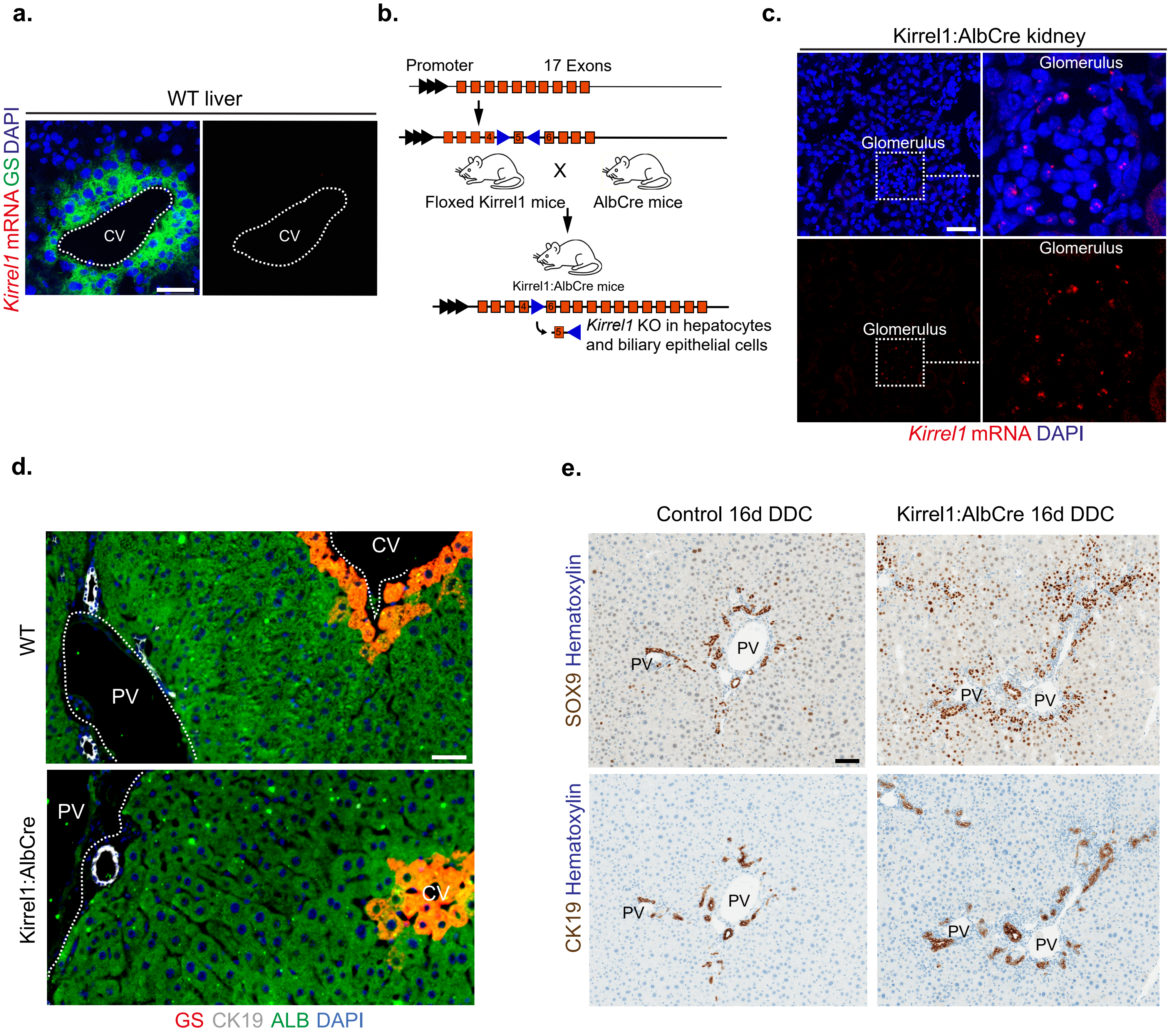

Figure S6. Generation and characterization of Kirrel1:AlbCre mice. a. *Kirrel1* ISH co-staining with the central vein (CV) hepatocyte marker Glutamine synthetase (GS) in naive WT mice. Scale bar, 100 μm. **b.** Scheme depicting generation of Kirrel1:AlbCre mice by crossing of mice with *Kirrel1* Exon5 flanked by LoxP sites (floxed Kirrel1 mice) with AlbCre mice, resulting in Exon5 deletion in hepatic epithelial cells. **c.** *Kirrel1* ISH staining in kidney glomeruli of Kirrel1:AlbCre mice. Scale bar, 100 μm. **d.** Co-staining for GS, CK19 (bile duct marker) and ALB (hepatocyte marker) in adult Kirrel1:AlbCre and WT mice indicates normal liver architecture and zonation despite *Kirrel1* deletion. PV, portal vein; CV, central vein. Scale bar, 50 μm. **e.** Exemplary SOX9 and CK19 IHC staining in control and Kirrel1:AlbCre mice with 16d DDC injury, used for quantifications in Fig. 6f and g, respectively (n=4 control and n=5 Kirrel1:AlbCre mice). PV, portal vein. Scale bar, 100 μm.

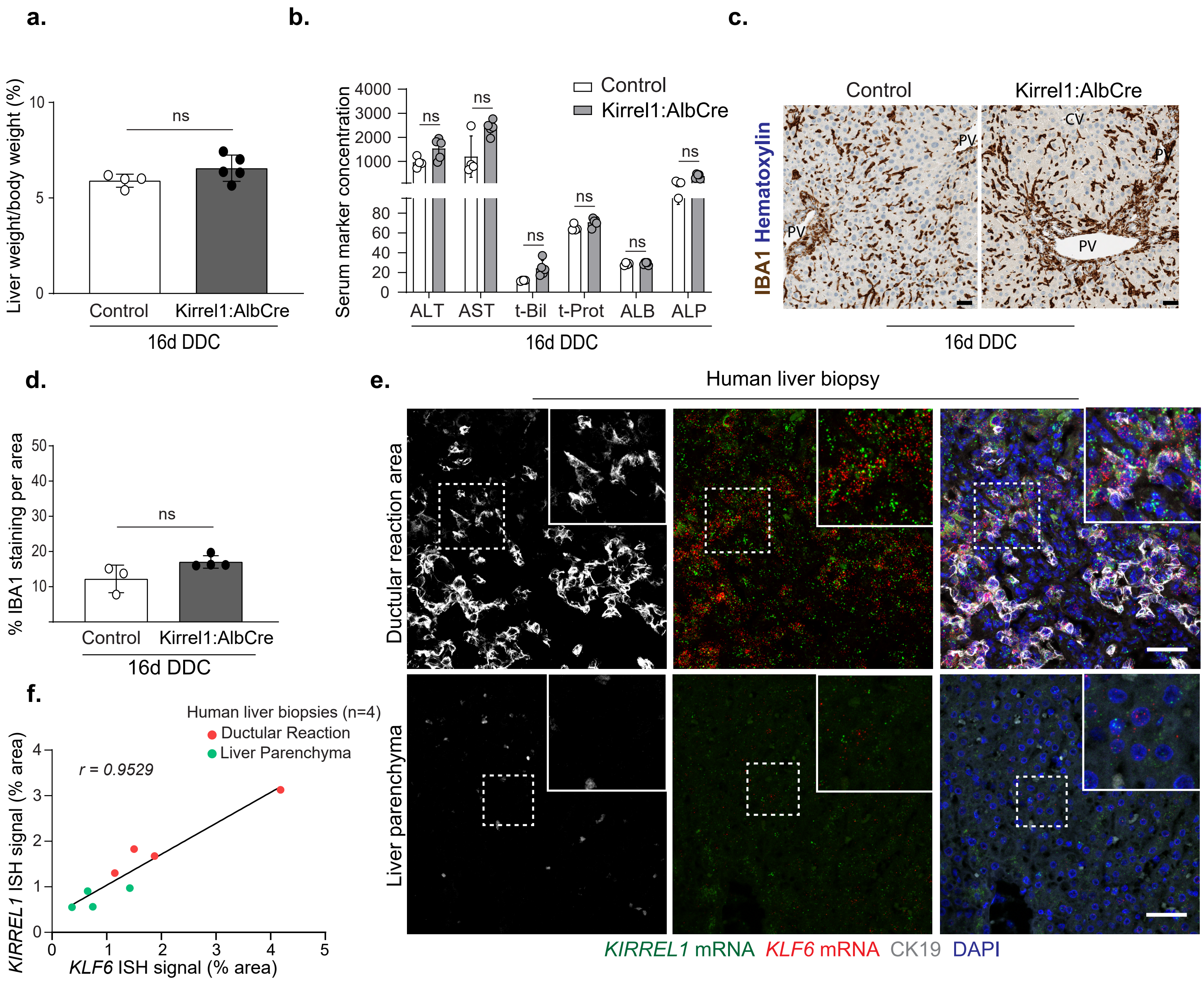

Figure S7. Damage assessment in DDC-injured mice and *KIRREL1* expression in human patient sample.
a. No difference in liver to body weight ratios following DDC diet in Kirrel1:AlbCre mice when compared to control mice. Data represent mean ± SD. ns, not significant; two-tailed unpaired *t*-test with groups of n=4 control and n=5 Kirrel1:AlbCre mice.
b. No significant differences in liver serum markers (ALT, alanine aminotransferase; AST,aspartate aminotransferase; t-Bil, total bilirubin; t-Prot, total protein, Alb, albumin; ALP, alkaline phosphatase) suggests similar DDC-induced liver injury in Kirrel1:AlbCre mice when compared to control mice. Data represent mean ± SD. ns, not significant; Multiple two-tailed unpaired *t*-tests with Welch correction and desired false discovery rate of 1% with groups of n=4 control and n=5 Kirrel1:AlbCre mice.
c, d. IBA1 (macrophage marker) staining (c) and quantification (d) suggest comparable inflammation in Kirrel1:AlbCre mice and controls following DDC diet. PV, portal vein; CV, central vein. Data represent mean ± SD; two-tailed unpaired *t*-test with n=3 control and n=4 Kirrel1:AlbCre mice. Scale bar, 50µm. **e.** ISH for *KIRREL1* and the YAP target gene *KLF6* on human liver sections (showing regions with either ductular reaction or liver parenchyma in the same patient). Scale bar, 50µm.
f. *KIRREL1* and *KLF6* ISH signals show high Pearson correlation (regions with either ductular reaction or liver parenchyma in 4 patients were quantified).

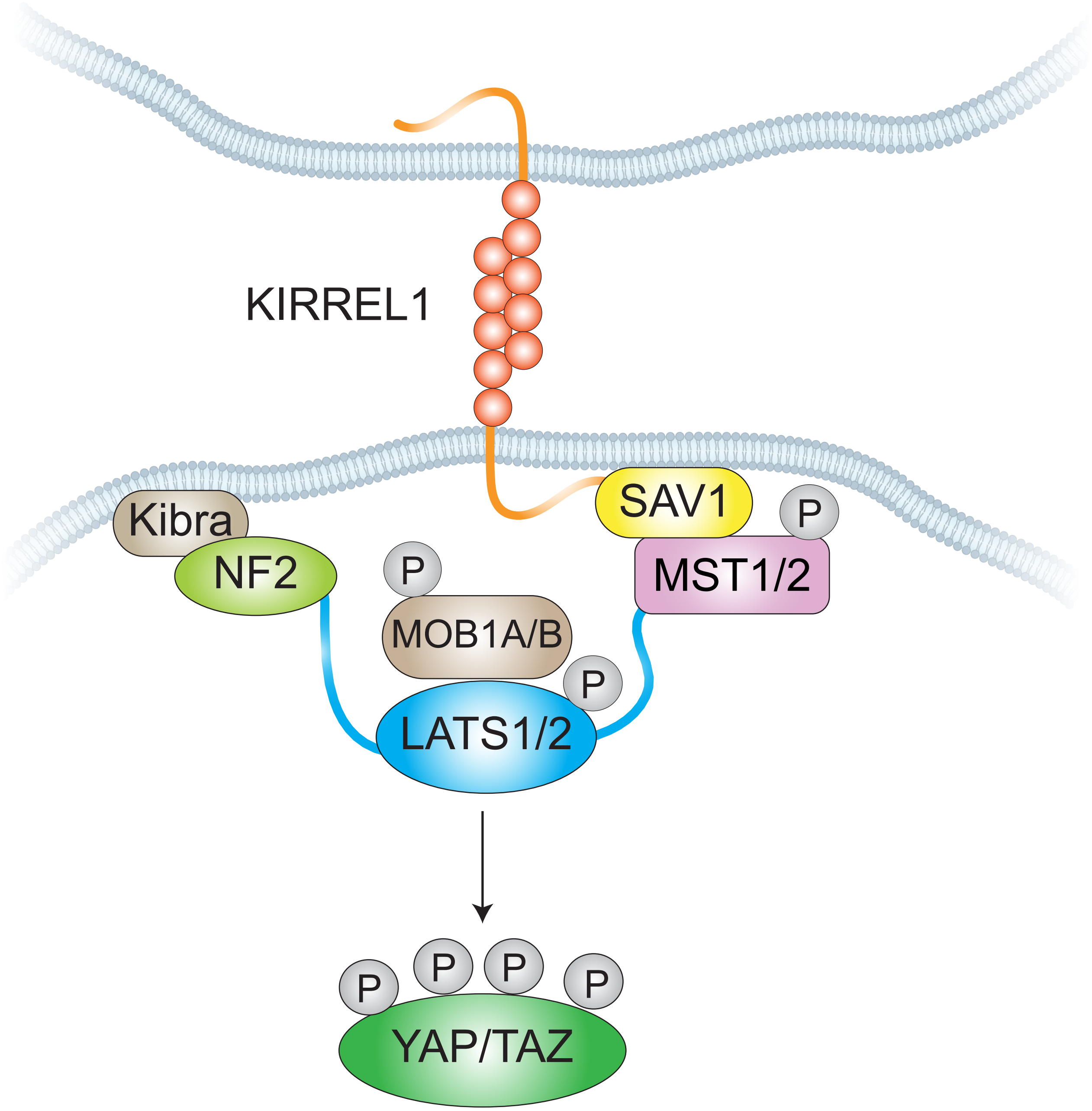

Figure S8. A schematic model of KIRREL1-mediated regulation of Hippo signaling

Gating strategy for Fig 1c

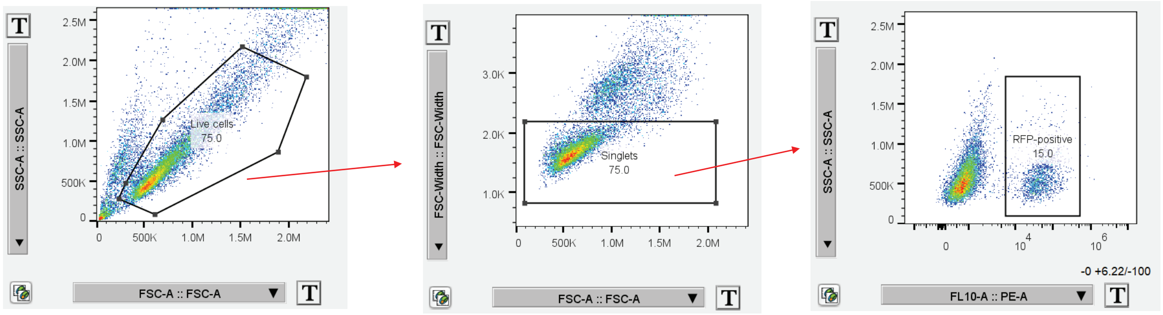

Gating strategy for Fig 1f

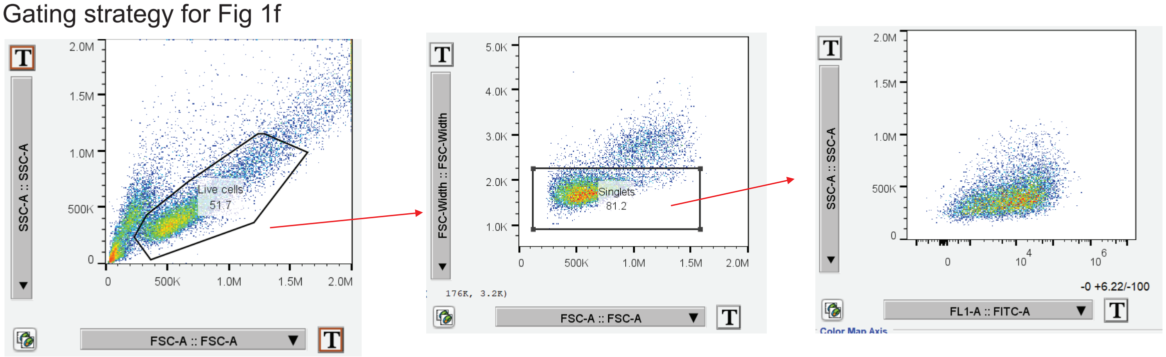

Gating strategy for Fig 2b

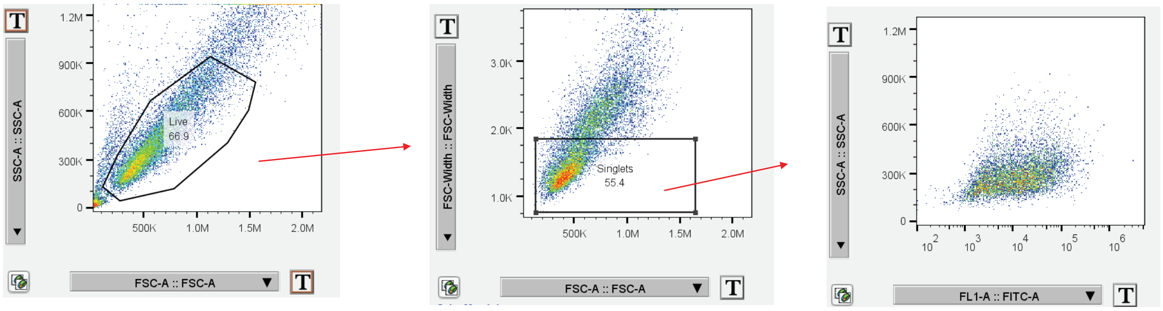

Gating strategy for Fig 4a

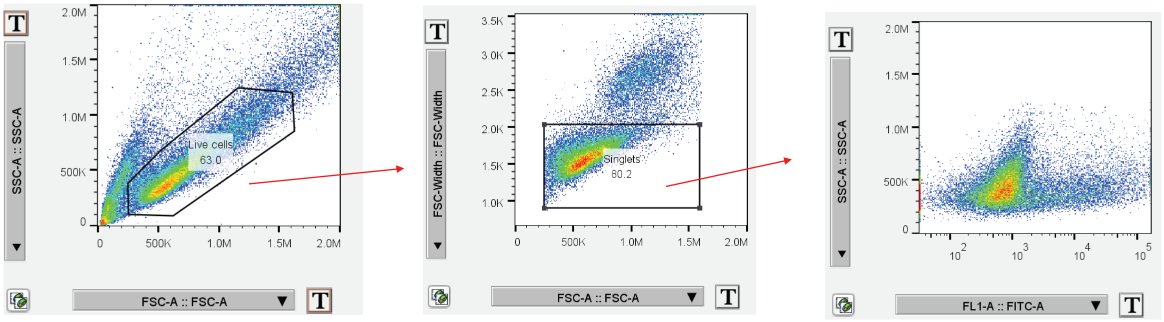

Gating strategy for Fig 4d

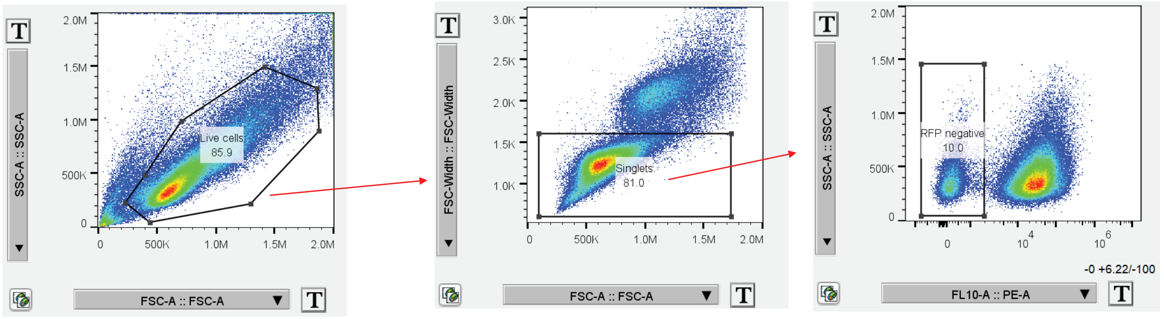

Figure S9. Gating strategy of FACS experiments.

Supplementary Tables

Supplementary Table 1: Oligo sequences used in gRNAs and shRNA

sgRNA	Sequence
Control (sgAAVS)	5’ GGGGCCACTAGGGACAGGAT
KIRREL1 gRNA1	5’ GAGTGAGGATCCAGACGAGG
KIRREL1 gRNA2	5’ GGAGCCAGCTGACCAGACGG
SAV1 gRNA –	5’ GAGCGAGAAGGACTTCCTC
YAP/TAZ gRNA –	5’ AATGTGGATGAGATGGATAC
**shRNA**	**Sequence**
Control shRNA	5’ CAACAAGATGAAGAGCACCAA
KIRREL1 shRNA1	5’ GGCCATCTACTCGTCGTTTAA
KIRREL1 shRNA2	5’ AACCTCACAAGACACAGGC

Supplementary Table 2: List of primers used in this study

Primer name	Primer sequence (5'-3')	Description
Full-length KIRREL1-F	AGCGTG GGATCC ATGGAGTGGGGTTACCTG	Cloning of full-length KIRREL1
Full-length KIRREL1-R	AGCGTG GCGGCCGC TGCGTAGTCTGG TACG	Cloning of full-length KIRREL1
KIRREL1- del ICD-F	AGCGTG GGATCC ATGGAGTGGGGTTACCTG	Cloning of KIRREL1 del ICD
KIRREL1- del ICD-R	AGCGTG GCGGCCGC TGCGTAGTCTGG TACG	Cloning of KIRREL1 del ICD
Kirrel1 del ICD1-F	5' PHOSPHO -TAGTAGCCATTGGTGGGGAGGAAGAATACCAAGG	Cloning of KIRREL1 del ICD1
Kirrel1 del ICD1-R	5 PHOSPHO-CCTTGGTATTCTTCCTCCCCACCAATGGCTACTA	Cloning of KIRREL1 del ICD1
Kirrel1 del ICD2-F	5' PHOSPHO-GTTCTCGTAGGACAGCTGGTCCTTCATCTCATACTC	Cloning of KIRREL1 del ICD2
Kirrel1 del ICD2-R	5' PHOSPHO -GAGTATGAGATGAAGGACCAGCTGTCCTACGAGAAC	Cloning of KIRREL1 del ICD2
Kirrel1 del ICD3-F	5'PHOSPHO-GGCACTGACACAACCAGCAAGAATTCTGCAGATATC	Cloning of KIRREL1 del ICD3
Kirrel1 del ICD3-R	5' PHOSPHO-GATATCTGCAGAATTCTTGCTGGTTGTGTCAGTGCC	Cloning of KIRREL1 del ICD3
His-SUMO-Kirrel1 ICD77 C-ter-F	GGTAGCGGCCGCAGCCAGCTGTCC	Cloning of KIRREL1 C-ter 77 Aa (pET-His-SUMO)
His-SUMO-Kirrel1 ICD77 C-ter-R	GGACAGCTGGCTGCGGCCGCTACC	Cloning of KIRREL1 C-ter 77 Aa (pET-His-SUMO)
His-SUMO-Kirrel1 ICD80 N-ter-F	CACGTTGTAGTAGCCATTGGTCTAGTCCTTCATCTCATACTCCTC	Cloning of KIRREL1 N-ter 80 Aa (pET-His-SUMO)
His-SUMO-Kirrel1 ICD80 N-ter-R	GAGGAGTATGAGATGAAGGACTAGACCAATGGCTACTACAACGTG	Cloning of KIRREL1 N-ter 80 Aa (pET-His-SUMO)
Flag-NF2-F	AGCCCGGGCGGGATCCGCCGGGGCCATCGCTTCC	Cloning of Flag-tagged NF2
Flag-NF2-R	CGGGCCCCCCCTCGAGCTAGAGCTCTTCAAAGAAGGCC	Cloning of Flag-tagged NF2
Flag-LATS1-F	AGCCCGGGCGGGATCCAAGAGGAGTGAAAAGCCAGAAG	Cloning of Flag-tagged LATS1
Flag-LATS1-R	CGGGCCCCCCCTCGAGCTAAACATATACTAGATCGCGATTT	Cloning of Flag-tagged LATS1
Flag-Mst1-F	AGCCCGGGCGGGATCCCTGTCCCGAAAGAAAACCAAAAACG	Cloning of Flag-tagged Mst1
Flag-Mst1-R	CGGGCCCCCCCTCGAGCTAAAAATTTTTTCCATGTTGTTGG	Cloning of Flag-tagged Mst1
Flag-MOB1-F	CGGGCCCCCCCTCGAGCTATCTGTCTTTTGATCCAAGTTTC	Cloning of Flag-tagged MOB1
Flag-MOB1-R	AGCCCGGGCGGGATCCGAGACGGTACAGCTGAGGAAC	Cloning of Flag-tagged MOB1
Flag-Kibra-F	AGCCCGGGCGGGATCCCCCCGGCCGGAGCTGCCC	Cloning of Flag-tagged Kibra
Flag-Kibra-R	CGGGCCCCCCCTCGAGTTAGACGTCATCTGCAGAGAGAGC	Cloning of Flag-tagged Kibra
Flag-Mst1-F	AGTGCG CTCGAGGAAGTTTTGTTGCCGTCTCTTC	Cloning of Flag-tagged Mst1
Flag-Mst1-R	AGTGCG GGATCCGCCGGGGCCATCGCTTCCCGC	Cloning of Flag-tagged Mst1
Flag-Sav1-F	AGTGCG GGATCCCTGTCCCGAAAGAAAACCAAAAAC	Cloning of Flag-tagged Sav1
Flag-Sav1-R	AGTGCG CTCGAGAAAATTTTTTCCATGTTGTTGGG	Cloning of Flag-tagged Sav1
GFP-SAV1-F	CTCAAGCTTCGAATTCATGCTGTCCCGAAAGAAAACCA	Cloning of GFP-tagged Sav1
GFP-SAV1-R	GGCGACCGGTGGATCCGCAAAATTTTTTCCATGTTGTTGGGCA	Cloning of GFP-tagged Sav1
Flag-Sav1_del1_F	GGAGGAAGGGGTTAATCTTCAGAACCATGGTTAGTC	Cloning of Flag-tagged del1 Sav1
Flag-Sav1_del1_R	GACTAACCATGGTTCTGAAGATTAACCCCTTCCTCC	Cloning of Flag-tagged del1 Sav1
Flag-Sav1_del2_F	TGATCATACCGAGGTACACTCTAAGCACAGGGATGCCTGTATTGG	Cloning of Flag-tagged del2 Sav1
Flag-Sav1_del2_R	CCAATACAGGCATCCCTGTGCTTAGAGTGTACCTCGGTATGATCA	Cloning of Flag-tagged del2 Sav1
Flag-Sav1_del3_F	CTGGAAGAGTTCCCACTTCAGCTAGTGGTCATATTTCACAGGGGC	Cloning of Flag-tagged del3 Sav1
Flag-Sav1_del3_R	GCCCCTGTGAAATATGACCACTAGCTGAAGTGGGAACTCTTCCAG	Cloning of Flag-tagged del3 Sav1
Flag-Sav1_del4_F	5'PHOSPHO CCGGGCGGGATCCTTACCCCTTCCTC	Cloning of Flag-tagged del4 Sav1
Flag-Sav1_del4_R	5'PHOSPHO GAGGAAGGGGTAAGGATCCCGCCCGG	Cloning of Flag-tagged del4 Sav1

Supplementary Table 3: Antibodies used in this study

Antibody	Company	Cat#	dilution
Anti-Flag	Sigma Aldrich	F1804	1 to 2000
Anti-Myc	CST	2278	1 to 1000
Anti-Myc	CST	2276	1 to 1000
Anti-HA	CST	3724P	1 to 1000
Anti-V5	CST	13202S	1 to 1000
anti-GAPDH	CST	8884S	1 to 1000
anti-SAV1	CST	3507S	1 to 1000
Anti-GFP	Thermo Fisher	A-11122	1 to 200
anti-Lamin A/C	CST	2032	1 to 1000
anti-YAP	CST	14074	1 to 1000
anti-YAP/TAZ	CST	8418	1 to 1000
anti p-YAP	CST	4911	1 to 1000
anti Histone H3	CST	4499	1 to 1000
Myc-Tag (9B11) Mouse mAb (Alexa Fluor® 488 Conjugate)	CST	2279	1 to 200
Goat Anti-Rabbit IgG Antibody, (H+L) HRP conjugate	Millipore Sigma	AP307P	1 to 5000
Goat Anti-Mouse IgG Antibody, (H+L) HRP conjugate	Millipore Sigma	AP308P	1 to 5000
