# Supplementary material for: Cell adhesion molecule KIRREL1 is a feedback regulator of Hippo signaling recruiting SAV1 to cell-cell contact sites
Source: Nat Commun. 2022 Feb 17;13:930. doi: 10.1038/s41467-022-28567-3 (PMC8854406; doi:10.1038/s41467-022-28567-3)
Supplement: Supplementary file 3 — Reporting Summary [file 41467_2022_28567_MOESM3_ESM.pdf]

nature portfolio

Corresponding author(s): Feng Cong

Last updated by author(s): Dec 10, 2021

# Reporting Summary

Nature Portfolio wishes to improve the reproducibility of the work that we publish. This form provides structure for consistency and transparency in reporting. For further information on Nature Portfolio policies, see our Editorial Policies and the Editorial Policy Checklist.

## Statistics

For all statistical analyses, confirm that the following items are present in the figure legend, table legend, main text, or Methods section.

| n/a | Confirmed | |
|---|---|---|
| ☐ | ☒ | The exact sample size (*n*) for each experimental group/condition, given as a discrete number and unit of measurement |
| ☐ | ☒ | A statement on whether measurements were taken from distinct samples or whether the same sample was measured repeatedly |
| ☐ | ☒ | The statistical test(s) used AND whether they are one- or two-sided<br>*Only common tests should be described solely by name; describe more complex techniques in the Methods section.* |
| ☒ | ☐ | A description of all covariates tested |
| ☒ | ☐ | A description of any assumptions or corrections, such as tests of normality and adjustment for multiple comparisons |
| ☐ | ☒ | A full description of the statistical parameters including central tendency (e.g. means) or other basic estimates (e.g. regression coefficient) AND variation (e.g. standard deviation) or associated estimates of uncertainty (e.g. confidence intervals) |
| ☐ | ☒ | For null hypothesis testing, the test statistic (e.g. $F$, $t$, $r$) with confidence intervals, effect sizes, degrees of freedom and $P$ value noted<br>*Give P values as exact values whenever suitable.* |
| ☒ | ☐ | For Bayesian analysis, information on the choice of priors and Markov chain Monte Carlo settings |
| ☒ | ☐ | For hierarchical and complex designs, identification of the appropriate level for tests and full reporting of outcomes |
| ☐ | ☒ | Estimates of effect sizes (e.g. Cohen's $d$, Pearson's $r$), indicating how they were calculated |

*Our web collection on statistics for biologists contains articles on many of the points above.*

## Software and code

Policy information about availability of computer code

| | |
|---|---|
| Data collection | Real-time PCR data was collected by software QuantStudio 7 Flex system, FACS data was collected by Cytoflex, BECKMAN COULTER Life Sciences. Luciferase data was collected by EnVision, PerkinElmer. |
| Data analysis | Statistics was analyzed by GraphPad Prism 8. The FACS data was analyzed by FlowJo V10. Raw sequencing reads from the CRISPR screen were aligned to the appropriate library using Bowtie, allowing for no mismatches, and counts were generated. The R software package DESeq2 was used to evaluate differential gRNA representation in the form of log2 fold change and P-value between the GFP-high and the GFP-low samples |

For manuscripts utilizing custom algorithms or software that are central to the research but not yet described in published literature, software must be made available to editors and reviewers. We strongly encourage code deposition in a community repository (e.g. GitHub). See the Nature Portfolio guidelines for submitting code & software for further information.

## Data

Policy information about availability of data

All manuscripts must include a data availability statement. This statement should provide the following information, where applicable:
- Accession codes, unique identifiers, or web links for publicly available datasets
- A description of any restrictions on data availability
- For clinical datasets or third party data, please ensure that the statement adheres to our policy

All relevant data supporting the findings of this study are available within the paper and Supplementary information files. Raw data and uncropped gel images of all figures are included in the Source Data file. We used the following publicly available datasets: the DepMap portal for dependency scores (https://depmap.org/portal) and GTEx database for tissue-specific gene expression (https://gtexportal.org/home/gene). All other data supporting the findings of this study are available from the corresponding author on reasonable request. Source data are provided with this paper.

# Field-specific reporting

Please select the one below that is the best fit for your research. If you are not sure, read the appropriate sections before making your selection.

- [x] Life sciences
- [ ] Behavioural & social sciences
- [ ] Ecological, evolutionary & environmental sciences

For a reference copy of the document with all sections, see nature.com/documents/nr-reporting-summary-flat.pdf

# Life sciences study design

All studies must disclose on these points even when the disclosure is negative.

| | |
|---|---|
| Sample size | Sample size is based on effect sizes from prior publications. Unless otherwise stated, experiments were carried out three or more times in three biologically independent samples. |
| Data exclusions | No data was excluded |
| Replication | All experiments were repeated at least three times and all attempts to replicate the experiments performed here were successful |
| Randomization | Sample allocation was random |
| Blinding | Data acquisition in this study analysis was conducted in a blinded manner. The investigators were blinded to group allocation during data collection to avoid conscious and unconscious bias. |

# Reporting for specific materials, systems and methods

We require information from authors about some types of materials, experimental systems and methods used in many studies. Here, indicate whether each material, system or method listed is relevant to your study. If you are not sure if a list item applies to your research, read the appropriate section before selecting a response.

## Materials & experimental systems

| n/a | Involved in the study | |
|---|---|---|
| | x | Antibodies |
| | x | Eukaryotic cell lines |
| x | | Palaeontology and archaeology |
| | x | Animals and other organisms |
| | x | Human research participants |
| x | | Clinical data |
| x | | Dual use research of concern |

## Methods

| n/a | Involved in the study | |
|---|---|---|
| x | | ChIP-seq |
| | x | Flow cytometry |
| x | | MRI-based neuroimaging |

## Antibodies

| | |
|---|---|
| Antibodies used | Anti-Flag tag (Cat# F1804, Sigma Aldrich), anti-Myc tag (Cat# 2278, CST), anti-Myc tag (Cat# 2276, CST),anti-HA tag (Cat# 3724P, CST), anti-V5 (Cat# 13202S, CST), anti-GAPDH (Cat# 8884S, CST), anti-SAV1 (Cat# 3507S, CST), anti-GFP (Cat# A-11122, Thermo Fisher), Histone H3 (Cat# 4499, CST), p-YAP (Cat# 4911, CST), YAP/TAZ (Cat#8418, CST), YAP (Cat#14074, CST), Lamin A/C (Cat# 2032, CST), Myc-Tag (9B11) Mouse mAb (Alexa Fluor® 488 Conjugate, Cat#2279, CST). Secondary antibodies conjugated with Alexa 488 (Cat# A-21206, Thermo Fisher) or Alexa 555 (Cat# A-31570, Thermo Fisher) were used for Immunofluorescence analysis at 1:1000 dilution. Goat Anti-Rabbit IgG Antibody, (H+L) HRP conjugate (Cat#AP307P,Millipore Sigma)<br><br>Dilution for each antibody is indicated in the Supplemental Table. |
| Validation | All primary antibodies used in this study are widely used and well validated in literature and by the manufacturer.<br><br>Anti-Flag antibody - https://www.sigmaaldrich.com/US/en/product/sigma/f1804<br>Anti-Myc tag antibody - https://www.cellsignal.com/products/primary-antibodies/myc-tag-antibody/2272.<br>Anti-Myc tag antibody - https://www.cellsignal.com/products/primary-antibodies/myc-tag-9b11-mouse-mab/2276<br>Anti-HA tag antibody - https://www.cellsignal.com/products/primary-antibodies/ha-tag-c29f4-rabbit-mab/3724<br>Anti-V5 antibody - https://www.cellsignal.com/products/primary-antibodies/v5-tag-d3h8q-rabbit-mab/13202?site-search-type=Products&N=4294956287&Ntt=13202s&fromPage=plp&_requestid=2414340<br>Anti-GAPDH antibody - https://www.cellsignal.com/products/antibody-conjugates/gapdh-d16h11-xp-rabbit-mab-hrp-conjugate/8884<br>Anti-SAV1 antibody - https://www.cellsignal.com/products/primary-antibodies/sav1-antibody/3507<br>Anti-GFP antibody - https://www.thermofisher.com/antibody/product/GFP-Antibody-Polyclonal/A-11122<br>Anti-Lamin A/C antibody - https://www.cellsignal.com/products/primary-antibodies/lamin-a-c-antibody/2032<br>Anti-YAP antibody - https://www.cellsignal.com/products/primary-antibodies/yap-d8h1x-xp-rabbit-mab/14074 |

Anti-YAP/TAZ antibody - https://www.cellsignal.com/products/primary-antibodies/yap-taz-d24e4-rabbit-mab/8418
Anti-phospho-YAP antibody - https://www.cellsignal.com/products/primary-antibodies/phospho-yap-ser127-antibody/4911
Anti-H3 antibody - https://www.cellsignal.com/products/primary-antibodies/histone-h3-d1h2-xp-rabbit-mab/4499
Myc-Tag (9B11) Mouse mAb (Alexa Fluor® 488 Conjugate) - https://www.cellsignal.com/products/antibody-conjugates/myc-tag-9b11-mouse-mab-alexa-fluor-488-conjugate/2279
Donkey anti-Rabbit IgG (H+L) Highly Cross-Adsorbed Secondary Antibody, Alexa Fluor 488 - https://www.thermofisher.com/antibody/product/Donkey-anti-Rabbit-IgG-H-L-Highly-Cross-Adsorbed-Secondary-Antibody-Polyclonal/A-21206
Donkey anti-Mouse IgG (H+L) Highly Cross-Adsorbed Secondary Antibody, Alexa Fluor 555 - https://www.thermofisher.com/antibody/product/Donkey-anti-Mouse-IgG-H-L-Highly-Cross-Adsorbed-Secondary-Antibody-Polyclonal/A-31570

# Eukaryotic cell lines

Policy information about cell lines

| | |
|---|---|
| Cell line source(s) | HEK293A (R70507, Thermo Fisher) and HEK293T (CRL-11268, ATCC) cells were obtained from the American Type Culture Collection. GAMG, DAOY, NCI-H2030, and IGR-39 cell lines were originated from the Cancer Cell Line Encyclopedia, banked at Novartis Cell Bank |
| Authentication | Cell lines obtained from Novartis Cell bank were authenticated by STR profiling. No extra authentication was performed for the cell lines. |
| Mycoplasma contamination | All cell lines tested negative for mycoplasma contamination |
| Commonly misidentified lines (See ICLAC register) | No commonly misidentified lines were used |

# Animals and other organisms

Policy information about studies involving animals; ARRIVE guidelines recommended for reporting animal research

| | |
|---|---|
| Laboratory animals | Mus musculus. 16-21 week old male C57Bl/6 mice were used for the experiments in this study. |
| Wild animals | No wild animals were used in the study. |
| Field-collected samples | No field collected samples were used in the study. |
| Ethics oversight | All animal experimentation was performed was conducted in accordance with animal law of and approved by the cantonal veterinary office Basel-Stadt, Switzerland. |

Note that full information on the approval of the study protocol must also be provided in the manuscript.

# Human research participants

Policy information about studies involving human research participants

| | |
|---|---|
| Population characteristics | For ISH on human livers, formalin-fixed paraffin-embedded (FFPE) sections of anonymized human needle liver biopsy tissue were obtained from the University Hospital Basel with ethics committee approval. Samples from 4 patients (aged 41-86; 3 males, 1 female) with liver injury-associated ductular reaction were used and analyzed in this study. |
| Recruitment | *Describe how participants were recruited. Outline any potential self-selection bias or other biases that may be present and how these are likely to impact results.* |
| Ethics oversight | The Formalin-fixed paraffin-embedded (FFPE) sections from human patients were obtained without compensation and used in accordance to the informed consent and approved by the ethics committee of northwest and central Switzerland (EKNZ). |

Note that full information on the approval of the study protocol must also be provided in the manuscript.

# Flow Cytometry

## Plots

Confirm that:

- [x] The axis labels state the marker and fluorochrome used (e.g. CD4-FITC).
- [x] The axis scales are clearly visible. Include numbers along axes only for bottom left plot of group (a 'group' is an analysis of identical markers).
- [x] All plots are contour plots with outliers or pseudocolor plots.
- [x] A numerical value for number of cells or percentage (with statistics) is provided.

## Methodology

| | |
|---|---|
| Sample preparation | Cells were harvested using cell dissociation buffer and resuspended in FACS buffer (PBS, 1% BSA), then subjected to Cytoflex cytometer. |
| Instrument | Cytoflex cytometer (Beckman Coulter Life Sciences) |
| Software | FlowJo |
| Cell population abundance | 10,000 cells |
| Gating strategy | Live cells were gated by FSC-A and SSC-A and singlets were derived from live cell population by FSC-Width and FSC-A. The RFP positive cells were gated where indicted. The GTIIC-GFP PEST signal was collected in FTIC channel. |

☒ Tick this box to confirm that a figure exemplifying the gating strategy is provided in the Supplementary Information.
